# Effect of prehabilitation exercises on postoperative frailty in patients undergoing laparoscopic colorectal cancer surgery

**DOI:** 10.3389/fonc.2024.1411353

**Published:** 2024-09-12

**Authors:** Fuyu Yang, Ye Yuan, Wenwen Liu, Chenglin Tang, Fan He, Defei Chen, Junjie Xiong, Guoquan Huang, Kun Qian

**Affiliations:** ^1^ Department of Gastrointestinal Surgery, The First Affiliated Hospital of Chongqing Medical University, Chongqing, China; ^2^ Department of Gastrointestinal Surgery, Central Hospital of Enshi Tujia and Miao Autonomous Prefecture, Enshi, China

**Keywords:** frailty, laparoscopic colorectal cancer surgery, colorectal cancer, enhanced recovery after surgery, prehabilitation exercises

## Abstract

**Background:**

To improve perioperative frailty status in patients undergoing laparoscopic colorectal cancer surgery (LCCS), we explored a new intensive prehabilitation program that combines prehabilitation exercises with standard enhanced recovery after surgery (ERAS) and explored its impact.

**Methods:**

We conducted a prospective randomized controlled trial. Between April 2021 to August 2021, patients undergoing elective LCCS were randomized into the standardized ERAS (S-ERAS) group or ERAS based on prehabilitation (group PR-ERAS). Patients in the PR-ERAS group undergoing prehabilitation exercises in the perioperative period in addition to standard enhanced recovery after surgery. We explored the effects of this prehabilitation protocol on frailty, short-term quality of recovery (QoR), psychological status, postoperative functional capacity, postoperative outcomes, and pain.

**Results:**

In total, 125 patients were evaluated, and 95 eligible patients were enrolled and randomly allocated to the S-ERAS (n = 45) and PR-ERAS (n = 50) groups. The Fried score was higher in the PR-ERAS group on postoperative day (7 (2(2,3) vs. 3(2,4), P = 0.012). The QoR-9 was higher in the PR-ERAS group than in the S-ERAS group on the 1st, 2nd, 3rd, and 7th postoperative days. The PR-ERAS group had an earlier time to first ambulation (P < 0.050) and time to first flatus (P < 0.050).

**Conclusion:**

Prehabilitation exercises can improve postoperative frailty and accelerate recovery in patients undergoing LCCS but may not improve surgical safety. Therefore, better and more targeted prehabilitation recovery protocols should be explored.

**Clinical trial registration:**

www.clinicaltrials.org **, identifier NCT04964856.**

## 1 Introduction

Frailty is a condition in which the body is unable to compensate and carries a high risk of disability, falls, hospitalization, and death. The core features of frailty include weakness, decreased endurance, and delayed performance (1). Patients with preoperative coexisting frailty had significantly more postoperative complications, longer lengths of hospital stay, and significantly slower recovery (2, 3). Frailty has been identified as a major obstacle to colorectal cancer (CRC) treatment in older patients (4).

Bed rest can cause muscle atrophy, insulin resistance, and disorders of fatty acid metabolism (5, 6). In addition, bed rest increases the risk of deep vein thrombosis and lung infections. Studies have confirmed that preoperative physical exercise effectively improves perioperative frailty (7, 8). Preoperative functional exercise can improve patients’ cardiopulmonary function and ability to cope with the stress of surgery and anesthesia, reduce the incidence of postoperative cardiovascular and cerebrovascular accidents, surgery-related complications (9, 10), and postoperative hospitalization time. In addition, preoperative functional exercise can improve patients’ postoperative survival rate and quality of life, and enhance patients’ postoperative regulation of blood fat and blood glucose (11).

Enhanced recovery after surgery (ERAS) is beneficial for early recovery after laparoscopic colorectal cancer surgery (LCCS), and reduces complications, readmission, and mortality (12, 13). However, its effectiveness does not appear to be significant (14), and a large systematic review concluded that ERAS does not reduce major complications after LCCS (15). CRC patients experience more psychological stress than general patients because they face the dual stress of surgery and cancer, therefore, surgeons should focus not only on the patient’s physical illness but also on their psychological state. Doctors can understand patients’ psychological status by assessing their anxiety, depression, and sleep status during hospitalization.

LCCS is one of the standard treatments for CRC. It requires intestinal resection and intestinal anastomosis, patients cannot eat normally immediately after surgery and need to combine enteral nutrition and parenteral nutrition to meet their nutritional needs, which not only prolongs the length of postoperative hospitalization and increases nutritional risk, but also deteriorates the patient’s hospital experience and increases psychological stress. A meta-analysis found that nutritional pre-rehabilitation alone or combined with an exercise significantly reduced the length of hospital stay for colorectal surgery patients (14). Another meta-analysis suggested that prehabilitation of colorectal cancer patients with postoperative recovery and reduced complication rates were beneficial (16). However, it is controversial whether it can improve postoperative functional capacity (17, 18), and the effect of either prehabilitation exercise or ERAS on postoperative prognosis is not satisfactory. Additionally, few studies have combined prehabilitation exercises and ERAS to examine their effects on postoperative outcomes. Both ERAS and prehabilitation exercises have been shown to improve the prognosis of patients undergoing LCCS. However, the main assessments have been length of hospital stay, postoperative complications, and functional recovery. To evaluate the effects of prehabilitation exercises combined with ERAS on postoperative vulnerability, psychological status, functional recovery, and postoperative complications in patients, we explored a new intensive prehabilitation protocol for prehabilitation exercises combined with standard ERAS. We also investigated the effects of this prehabilitation protocol on frailty, short-term recovery quality, psychological status, postoperative functional capacity, postoperative outcomes, and pain.

## 2 Patients and methods

### 2.1 Study design

This single-center, prospective, randomized controlled trial was conducted at the First Affiliated Hospital of Chongqing Medical University between April 2021 and August 2021. To report this trial, we adhered to the Consolidated Standards of Reporting Trials Statement (CONSORT). Patient enrolment was initiated after written informed consent was obtained. This study was reviewed and approved by the Medical Ethics Committee of Chongqing Medical University.

### 2.2 Patients

Patients were recruited from a treatment group at the Department of Gastrointestinal Surgery. Inclusion criteria for the patients were as follows: Patients undergoing elective LCCS, aged >18 years and ≤100 years, with an American Society of Anesthesiologists (ASA) score is between I and III, who could clearly understand and voluntarily participate in the study and sign an informed consent form. Exclusion criteria were as follows: Emergency surgery; those with a history of cognitive dysfunction; an ASA score ≧̸IV; those with a history of spontaneous pneumothorax, coagulation dysfunction, acute and systemic infectious diseases, and moderate or higher fever; pregnant women; those with a history of drug abuse; those who were judged by the physician in charge to be unsuitable for ERAS-exercise; those with other severe cardio-pulmonary diseases that would affect the 6-minute walking distance (6MWD); and those who failed to provide informed consent.

### 2.3 Sample size calculation

The sample size was estimated based on the difference in frailty between the two groups at discharge. The mean population difference between the experimental and control groups was expected to be 0.6 with a standard deviation of 0.8. It was assumed that the ERAS exercise group had a better attenuation of frailty than in the control group. The test level (α) for this parameter was set at 0.050, and a two-sided two-sample unequal variance t-test was used with β = 0.9. As the experimental group: control group ratio was 1:1, with 38 cases required for each group, 48 cases in each of the experimental and control groups were selected to account for a 20% shedding rate. Allocation was performed in the preoperative clinic. The diagnosis was confirmed on the day of admission and surgery was scheduled. This study used a simple randomization method. A random sequence was generated by a random number table generated by SPSS software (version 25.0), odd numbers were assigned to the PR-ERSA group and even numbers to the S-ERSA group. The table of random numbers is kept by statisticians independent of the research team and inaccessible to researchers before and during grouping. The grouping information was sealed in opaque and sequentially coded envelopes, and it was not until the subjects met the inclusion criteria and decided to participate in the study that the grouping was revealed by this statistician who opened the envelopes on the spot in the presence of the researcher and the subjects.

### 2.4 Patient characteristics and perioperative management

The following information was collected from the patients before surgery: Sex, age, body mass index (BMI), history of smoking and drinking, occupation, education level, preoperative hemoglobin and albumin levels, preoperative diagnosis, preoperative American Society of Anesthesiologists (ASA) grading, New York Heart Association (NYHA) grading, Nutritional Risk Screening 2002 (NRS-2002) score, and activities of daily living (ADL) scores. We discussed the surgical plan for each patient the day before surgery and then performed a colectomy or rectectomy according to the scheduled operation plan. We used a midline incision for specimen extraction from the right colon lesions, a transverse incision for the left colon lesions, and a left lower abdominal oblique incision for the rectal lesions. In rectal resection, if the risk of anastomotic leakage was high, we performed a temporary ileostomy and reversed the ileostomy 2-3 months later. No nasogastric tube was inserted, and abdominal drainage was not routinely placed unless the risk of anastomotic leakage high. After patient-controlled intravenous analgesia (PCIA) removal, the patient may receive additional non-selective NSAIDs (flurbiprofen ester) if necessary.

After informed consent was obtained and surgery was scheduled, patients were randomized into the PR-ERAS and standardized enhanced recovery after surgery (S-ERAS) groups. For patients in the PR-ERAS group, prehabilitation exercises twice a day based on a standardized enhanced prehabilitation protocol for the S-ERAS group (**Supplementary Material 1**) were designed with formatted exercise instructions.

### 2.5 Prehabilitation exercises

Once the patient’s elective surgery was scheduled, a rehabilitation therapist provided the patient with details of the exercise on a video. The patients performed prehabilitation exercises at home if they could not be temporarily admitted for surgery, and once they were admitted to the hospital, they performed prehabilitation exercises at the bedside. The prehabilitation exercise lasted until the day before the operation. Since the patients in this study were all cancer patients, in order to avoid the risk of cancer progression increased by long waiting time, the colorectal cancer surgery was completed as scheduled, and the duration of preoperative prehabilitation exercise was not extended. The exercise program consisted of three elements: Upper limb exercises, breathing exercises, and lower limb exercises (**Table 1**). The prehabilitation exercises include slow, non-weight-bearing movements of the limbs, thorax and abdomen, each lasting 10 to 15 minutes. These exercises do not cause a large increase in heart rate and respiratory rate, so they are light-intensity.

**Table 1:** Prehabilitation exercise regimens.

Exercise programs	Types of Movement
Upper limb exercises	Hands squeeze and relaxation
	Elbow flexion and extension
	Arms raised flat in front and spread upward
	Horizontal elbow flexion and chest expansion, then spread arms
Breathing exercises	Deep inhalation to expand the thorax, lip reduction and exhalation
	Deep inhalation to expand the abdomen, slow exhalation
	Deep inhalation, exhale 3 times, expectorate
Lower limb exercises	Ankle flexion and extension
	Ankle rotation
	Quadriceps contraction and relaxation

Patients in the PR-ERAS group were encouraged to perform this exercise twice, in the morning and afternoon, with 10-15 repetitions of each movement. The rehabilitation therapist provided each patient with a diary to record the exercise duration and type. The rehabilitation therapist advised each patient to follow the exercise program and supervised their exercise through WeChat or phone every day. Therefore, the light-intensity exercise was performed under close supervision and was individualized according to each patient’s tolerance. The patients were also encouraged to perform postoperative rehabilitation exercises with the help of a rehabilitation therapist. If the patient complained of any discomfort, such as a heart rate >100/min, severe weakness, chest tightness, or dizziness, the prehabilitation exercise program was suspended and a rehabilitation therapist was consulted to decide whether to resume prehabilitation exercises based on the patient’s condition. **Supplementary Figure S1** shows details of the prehabilitation exercises.

### 2.6 Outcome assessments

The primary outcome of this study was the Fried’s Frailty Phenotype (FP) scores(1), the scores from 0 to 5 (1 point for each component; 0 = best to 5 = worst) and represent robust (0), prefrail (1-2), and frail (3-5) health status. It include following five components: (1) unintentional weight loss of ≥4.5 Kg or ≥5% of body weight in the prior year; (2) slowness, with the slowest 20% of the population walking 5 meters, adjusting for gender and standing height; (3) weakness, with dominant grip strength in the lowest 20% at baseline, adjusted for gender and body mass index; (4) low physical activity level, according to the Minnesota leisure time physical activity questionnaire, with a weighted score of the kilocalories consumed per week calculated for each participant, and (5) poor endurance and energy according to self-reported exhaustion, identified by two questions from the CES–D scale. The FP scores were assessed on the day of the elective surgery and on the seventh postoperative day. Secondary outcomes included perioperative functional capacity, which was assessed using the 6-minute walking distance (6MWD) (19) test on the day of scheduled surgery and on postoperative day 7. In addition, we measured the Borg Dyspnea Scale score before and after each test to assess the degree of dyspnea (20). The quality of recovery (QoR-9) score (21) evaluated both physical and mental well-being by assessing five dimensions: Emotional state, physical comfort, psychological support, physical independence, and pain at the first, second, third, and seventh postoperative days. The patient’s mood state was assessed using the Hospital Anxiety and Depression Scale (HADS) questionnaire (22). The HADS is a fourteen items scale, with seven of the items related to anxiety and seven to depression. It was assessed on the day of surgery and seven days after surgery. Patient sleep status on the scheduled day of surgery and on the seventh postoperative day was assessed using the Sleep Self-Rating Scale (SRSS). The Visual Analog Pain Scale (VAS) is a unidimensional measure of pain intensity and consists mainly of a 100 mm straight line (23). The patient marked the corresponding position on this line. Each patient was asked to provide the VAS scores at rest and during exercise at 24 hours, 48 hours, 72, hours and 7 days postoperatively. Before surgery, we assessed the patients’ nutritional status and ability to live using the Nutritional Risk Screening 2002 (NRS-2002) (24) and Activities of Daily Living Scale (ADL), respectively.

Postoperative pneumonia and surgical complications (anastomotic leak, surgical site infection, incision infection, hemorrhage, lymphorrhea, ileus, and gastroparesis) were the secondary outcomes. Recovery parameters, such as time to ambulation, time to pass gas, time to first defecation, time to fluid intake, time to pull out the catheter, postoperative hospital stay, reoperation, and readmission, were compared between the two groups.

### 2.7 Statistical methods

SPSS software (version 25.0; IBM Corporation, Armonk, N.Y., USA) was used for the statistical analysis. Continuous variables were expressed as mean ± standard deviation and range (interquartile range), according to the normality of the data. The Kolmogorov-Smirnov test was used to determine the normal distribution of continuous variables. Continuous variables were compared using the independent Student’s t-test or Mann–Whitney U test, depending on the normality of the data. Categorical variables, such as sex, ASA Grading, type of surgery, and presence and incidence of complications, were expressed as frequencies and percentages. The chi-squared test was used to compare categorical variables. Statistical significance was set at P < 0.05.

## 3 Results

### 3.1 Basic characteristics and postoperative pain

**Figure 1:**
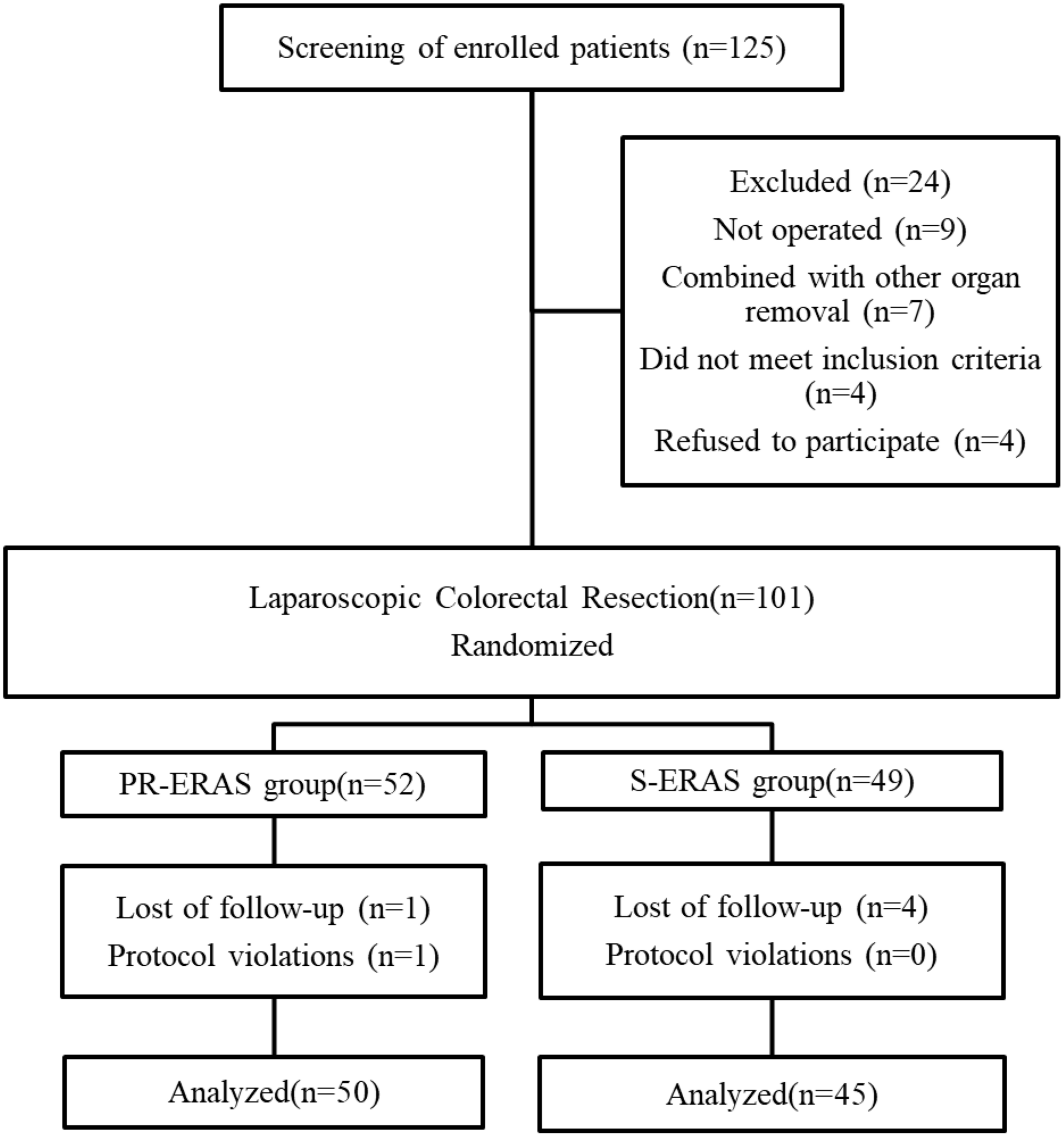
Study flow diagram. ERAS, Enhanced recovery after surgery; PR-ERAS, Enhanced recovery after surgery based on prehabilitation; S-ERAS, Standardized enhanced recovery after surgery.

Between May 2021 and December 2021, 125 patients were evaluated for inclusion in the study, following the process shown in **Figure 1**. A total of 24 patients were excluded because they either did not meet the inclusion criteria or refused to participate. During the trial, one patient in the PR-ERAS group was lost to follow-up before admission and one patient did not exercise as planned, which was considered a protocol violation. Four patients in the S-ERAS group were lost, and 95 patients were included and randomized into the PR-ERAS (n = 50) and S-ERAS (n = 45) groups. The duration of prehabilitation exercise in the PR-ERAS group was 13.2 ± 4.8 days. None of the patients in the PR-ERAS group had their surgery delayed or cancelled because of exercise, and no injuries or falls occurred. The increase in walking time (s) (1.55(0.9,2.1) vs. 2(1.6,2.8), P=0.011) of the PR-ERAS group was shorter than that of the S-ERAS group, and the drop in grip strength (Kg) (3.35(1.8,5.7) vs. 5.5(4.2,8.7), P<0.001) decreased less, and the difference was statistically significant. But the difference in other baseline clinical variables between the two groups was not statistically significant (**Table 2**). No statistical differences were found in the postoperative pain between the two groups (**Table 3**). No patients converted to open surgery, and there was no preoperative pain.

**Table 2:** Baseline clinical variables.

	PR-ERAS group (n=50)	S-ERAS group (n=45)	P
Age (year)	60 ± 10	64 ± 12	0.084
Male	34 (68)	24 (53.3)	0.143
BMI (Kg/m^2^)	22.7 ± 2.4	23.0 ± 2.9	0.644
Smoking	26 (52)	16 (35.6)	0.107
Drinking	22 (44)	14 (31.1)	0.196
Preoperative hemoglobin (g/L)	128 ± 20	124 ± 23	0.452
Preoperative albumin (g/L)	42 ± 4	41 ± 6	0.193
ASA Grading (I/II/III)	1/31/18	3/19/23	0.126
NYHA Grading (I/II/III)	5/43/2	3/33/9	0.054
TNM stage (I/II/III/IV)	13/26/19/2	12/20/10/3	0.854
From enrolment to admission (day)	9 ± 4.6	7.9 ± 2	0.154
From admission to surgery (day)	4.3 ± 1.8	4.9 ± 2.7	0.147
From enrolment to surgery (day)	13.2 ± 4.8	12.8 ± 3.2	0.638
Highly educated	7 (14)	9 (20)	0.435
Main comorbidity^a^	19 (38)	19 (42.2)	0.675
No. of daily medication	0 (0,1)	0 (0,1)	0.214
Type of colectomy (right/left/sigmoid/rectal)	8/3/5/34	11/7/6/21	0.173
Ostomy	17 (50)	7 (33.3)	0.226
Laparoscopic surgery	50 (100%)	45 (100%)	NA
Hospitalization expenses	70293 (64010,75526)	72067 (68691,78733)	0.061
NRS2002	3 (2,4)	3 (2,4)	0.681
ADL	100 (100,100)	100 (100,100)	NA
Increase in walking time (s)	1.55 (0.9,2.1)	2 (1.6,2.8)	0.011
Drop in grip strength (Kg)	3.35 (1.8,5.7)	5.5 (4.2,8.7)	<0.001

Variables are presented as n (%), mean ± standard deviation, or median (interquartile range). PR-ERAS, enhanced recovery after surgery based on prehabilitation; S-ERAS, Standardized enhanced recovery after surgery; BMI, body mass index; ASA, American Society of Anesthesiologists; NYHA, New York Heart Association; NRS-2002, Nutritional Risk Screening 2002; ADL, activities of daily living. ^a^Include hypertension, diabetes mellitus, coronary atherosclerotic heart disease, chronic obstructive pulmonary disease, cerebral infarction, and asthma. NA, Not Applicable.

**Table 3:** Postoperative pain.

	PR-ERAS group (n=50)	S-ERAS group (n=45)	P
VAS at rest			
24 hours after surgery	0 (0,1)	0 (0,1)	0.929
48 hours after surgery	0 (0,1)	0 (0,1)	0.455
72 hours after surgery	0 (0,0)	0 (0,0)	0.264
the day of discharge	0 (0,0)	0 (0,0)	0.134
VAS during exercise			
24 hours after surgery	2 (2,3)	2 (2,3)	0.737
48 hours after surgery	2 (1,2)	2 (2,2)	0.216
72 hours after surgery	1.5 (1,2)	2 (1,2)	0.093
the day of discharge	1 (1,1)	1 (1,1)	0.928
Number of PCIA presses	43.1 ± 11	44.9 ± 10.9	0.438
Dosage of PCIA (mL)	2 (1,5)	3 (1,5)	0.905
Rescue analgesia	15 (30)	13 (28.8)	0.906

Variables are presented as n (%), mean ± standard deviation, or median (interquartile range). VAS, Visual Analog Pain Scale; PCIA, patient-controlled intravenous analgesia.

### 3.2 Fried’s frailty phenotype scores and frailty status

**Figure 2:**
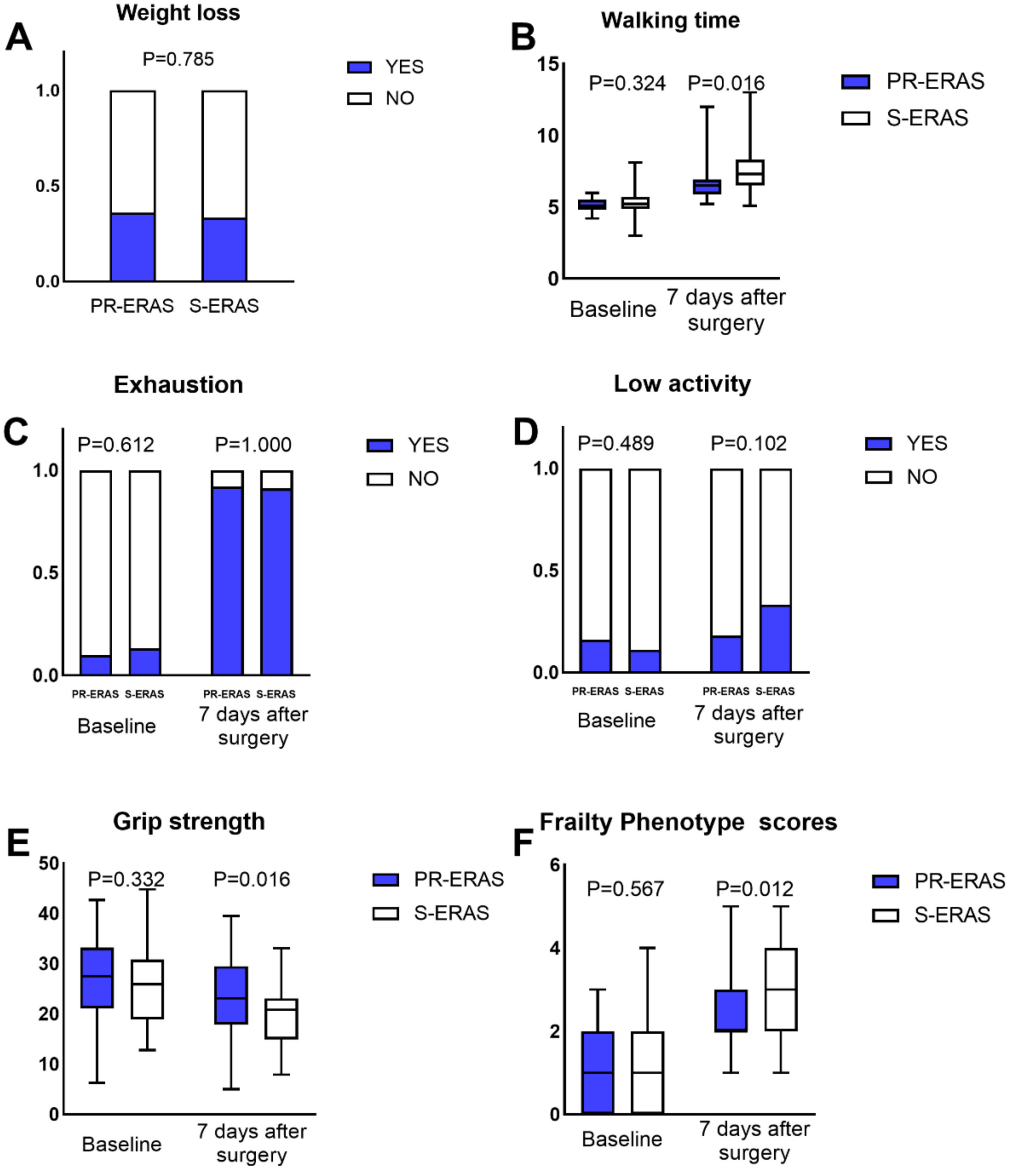
Fried’s Frailty Phenotype (PF) scores and its five criteria. The walking time, grip strength and PF scores expressed as the median (range), the weight loss, exhaustion and low activity expressed as percentages. Only the differences in walking time, grip strength and PF scores were statistically significant, the others were statistically similar. ERAS, Enhanced recovery after surgery. **(A)** Weight loss; **(B)** Walking time; **(C)** Exhaustion; **(D)** Low activity; **(E)** Grip strength; **(F)** Frailty Phenotype scores.

Before the scheduled surgery, the frailty score, frailty status, and five criteria were similar between the two groups. On postoperative day 7, the difference in the FP scores between the PR-ERAS and S-ERAS groups was statistically significant (P=0.012), with median and quartiles of 2(2,3) and 3(2,4), respectively. Among its five criteria, only the walking time (s) (6.5(5.9,6.9) vs. 7.3(6.7,8.3), P=0.016) and grip strength (Kg) (23.1 ± 7.5 vs. 19.6 ± 6.6, P=0.016) were smaller in the PR-ERAS group than in the S-ERAS group, and the differences were statistically significant, while the others were statistically similar (**Figure 2**).

### 3.3 Short-term recovery quality and psychosocial status

**Figure 3:**
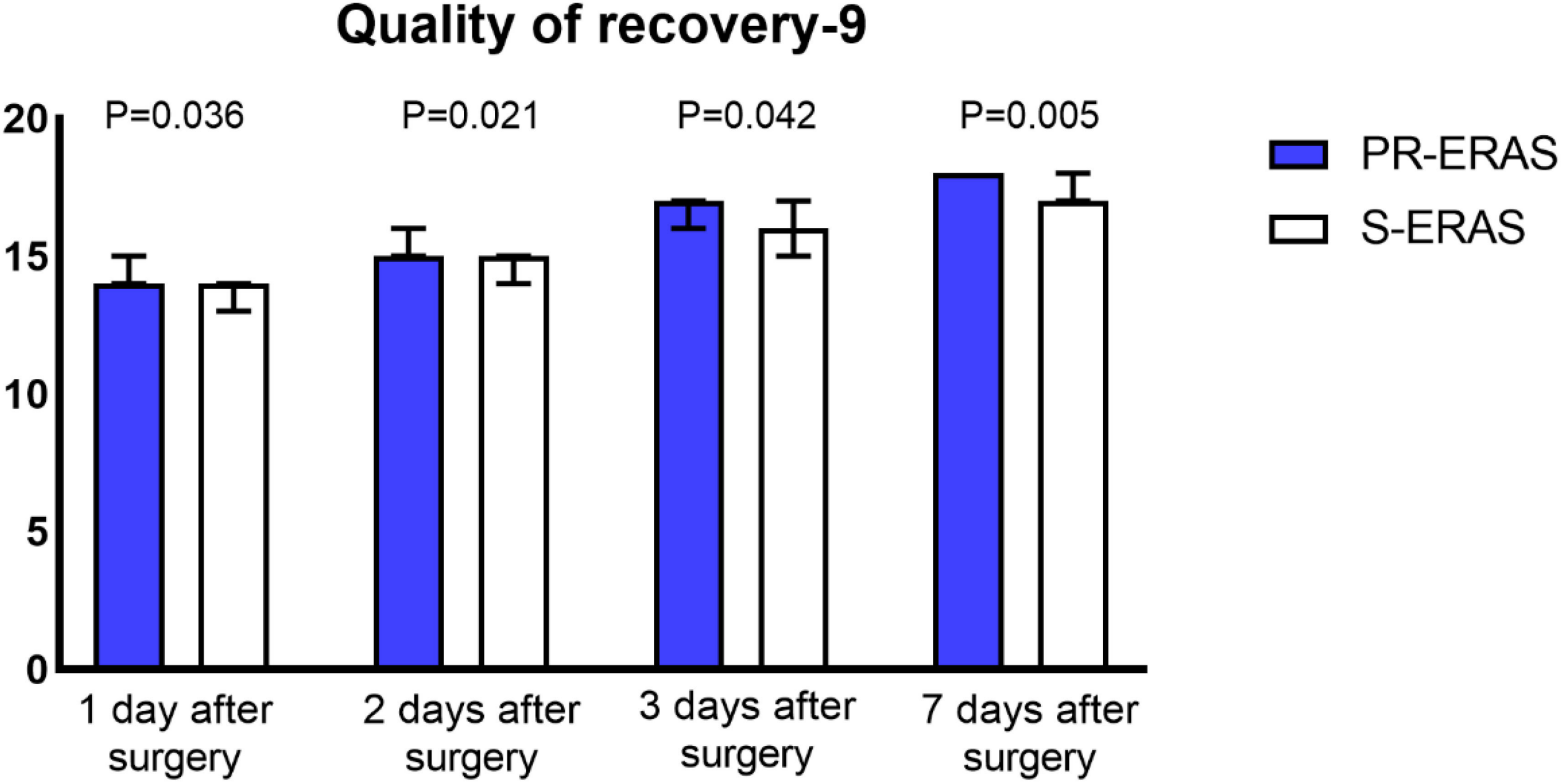
Quality of recovery (QoR-9) scores. The differences in all four comparisons were statistically significant.

**Figure 4:**
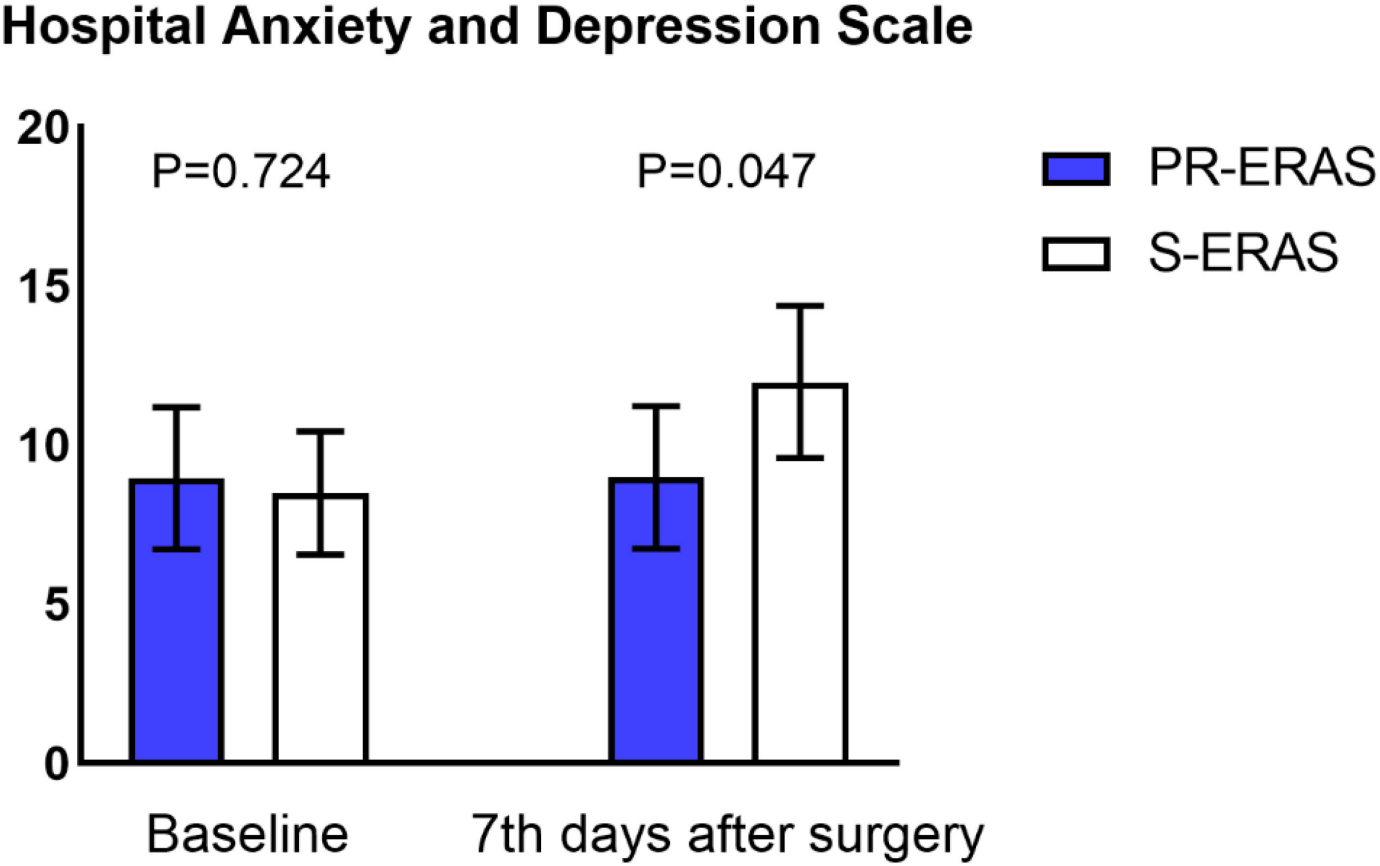
Hospital Anxiety and Depression Scale (HADS). Only the difference on postoperative day 7 was statistically significant.

**Figure 5:**
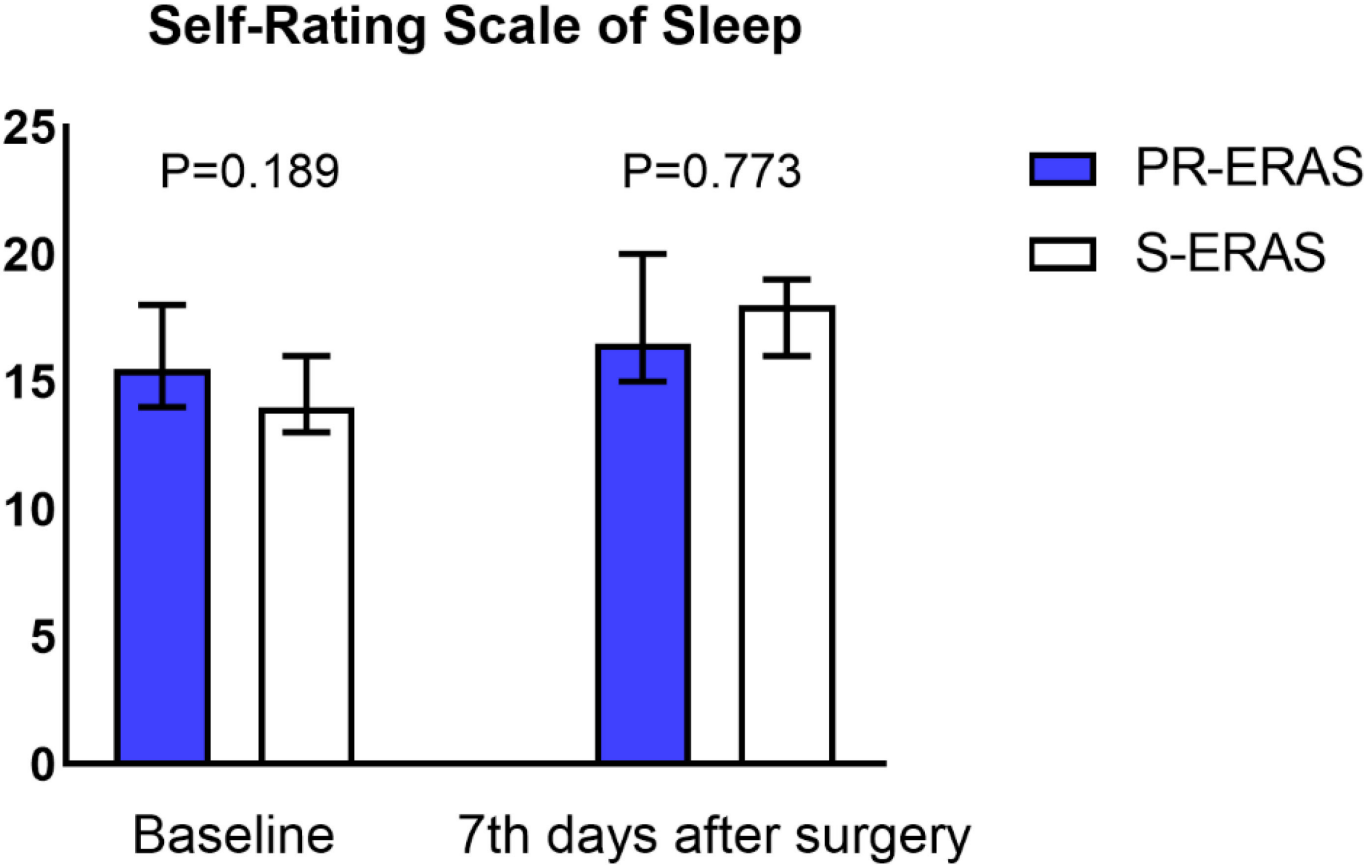
Sleep Self-Rating Scale (SRSS). The differences in both assessments were not statistically significant.

Prehabilitation exercises had significant improvement on postoperative recovery. The QoR-9 scores in the S-ERAS group on the first postoperative day (14(13,16) vs. 14(13,14) P=0.036), second day (15(14,16) vs. 15(13,16) P=0.021), third day (17(15,17) vs. 16(15,17) P = 0.042), and seventh day (18(17,18) vs. 17(16,18) P = 0.005) were all better than in the S-ERAS group, and the differences were statistically significant (**Figure 3**). The preoperative sleep and psychological status were similar between the groups. However, postoperative HADS scores were lower in the PR-ERAS group than in the S-ERAS group (p=0.047), and the median and interquartile ranges for the two groups were 2 (2–15) and 14(4–17), respectively (**Figure 4**). However, the difference in SRSS scores between the two groups was not statistically significant (**Figure 5**).

### 3.4 Perioperative functional capacity

The PR-ERAS and S-ERAS groups showed similar preoperative and postoperative 6MWD results. We performed Brog scoring before and after both trials, and only the PR-ERAS group had a lower Brog score after 6MWD postoperatively than in the S-ERAS group (0.5(0,1) vs. 1(0.5,1), respectively, P=0.028), which was a statistically significant difference, while the results were similar at all other times (**Table 4**).

**Table 4:** 6MWD and borg score when scheduled surgery and 7 days after surgery.

	PR-ERAS group (n=50)	S-ERAS group (n=45)	P
When scheduled surgery			
6MWD	430 (400,500)	420 (335,480)	0.124
Borg score before 6MWD	0 (0,0)	0 (0,0)	0.134
Borg score after 6MWD	0 (0,0.5)	0 (0,0.5)	0.091
7 days after surgery			
6MWD	267.5 (230,300)	250 (180,300)	0.191
Borg score before 6MWD	0.5 (0,1)	1 (0.5,1)	0.028
Borg score after 6MWD	0 (0.5,1)	0 (0.5,1)	0.426

6MWD, 6-minute walking distance.

### 3.5 Surgical outcomes and postoperative complications

Compared to S-ERAS, the PR-ERAS group had earlier time to first ambulation (Hour) (13(10,15) vs. 15(12,20) P=0.006) and time to first flatus (Hour) (14(8.5,25) vs. 18.5(14,40) P=0.039). The rates of intraoperative blood loss, intraoperative blood transfusion, postoperative pneumonia, and other complications were statistically similar between the groups (**Table 5**).

**Table 5:** Surgical outcomes.

	PR-ERAS group (n=50)	S-ERAS group (n=45)	P
Intraoperative blood loss (mL)	30 (20,50)	2 (20,50)	0.496
Intraoperative blood transfusion	0	2 (4.4)	0.222
Operation time (min)	154 (140,170)	150 (134,170)	0.383
Time to ambulation (h)	13 (10,15)	15 (12,20)	0.006
Time to first flatus (h)	14 (8.5,25)	18.5 (14,40)	0.039
Time to first defecation (h)	26.5 (10.5,40)	38 (16,60)	0.121
Time to fluid intake (h)	4 (4,4)	4 (4,4)	0.948
Time to pull out urinary catheter (h)	36 (19,61)	37.5 (19,59)	0.702
Admitted to ICU	0	3 (6.6)	0.103
Pneumonia	1 (2)	3 (6.6)	0.342
Surgical complications^a^	7 (14)	9 (20)	0.435
Postoperative hospital days (d)	5 (4,6)	5 (4,7)	0.363
Reoperation	3 (6)	1 (2.2)	0.619
Readmission within one month	3 (6)	3 (6.6)	1
Death within one month	0	1 (2.2)	0.474

ICU, Intensive care unit. ^a^Include anastomotic leak, hemorrhage, lymphorrhea, ileus, gastroparesis, incision infection, and abdominal infection.

### 3.6 Subgroup analysis of PR-ERAS group

Considering the difference in surgical procedures (colectomy and proctectomy) and the different ostomy rates in PR-ERAS group, subgroup analysis was performed in the PR-ERAS group. The NRS2002 score, PF score, 5 criteria in PF score, HADS score, SRSS score, QoR-9 score, 6MWD score and borg score were compared between different groups of patients. There is no statistical difference was observed in subgroup analysis. (**Supplementary Tables S1**, **S2**)

## 4 Discussion

Based on the results of this study, we suggest that adding prehabilitation exercises to a standardized enhanced recovery program may further improve postoperative frailty status, increase the quality of short-term postoperative recovery, accelerate postoperative recovery, and partially improve postoperative psychosocial status. However, it may not improve postoperative functional capacity or reduce the incidence of postoperative complications.

To our knowledge, no study has evaluated the effect of prehabilitation exercises combined with ERAS on frailty status after LCCS. Physical exercise may lead to inadequate gastrointestinal perfusion and gastrointestinal damage and symptoms such as nausea, vomiting, and abdominal pain (25). Therefore, we monitored the patients for gastrointestinal discomfort during the preoperative exercise. Fortunately, no gastrointestinal discomfort was reported among the patients who followed the prehabilitation exercises, nor did anyone suffer from complications such as falls and injuries. This suggests that prehabilitation exercises are safe and effective for improving frailty and enhancing the quality of short-term recovery after LCCS. Our prehabilitation protocol is easy, and there was a video to guide patients, they can do it alone. The main role of rehabilitation therapists is to supervise patients to complete prehabilitation exercises and corrective actions, so the patient costs little.

FP scores consist of shrinking, weakness, poor endurance and energy, slowness, and low physical activity level, where patients without features were considered robust, meeting 1-2 features was defined as prefrail, and those with three or more critical features were considered vulnerable (1). Frailty is reversible and can be improved with reasonable interventions. Previous studies have shown promising effects on frailty status through trials of non-pharmacological interventions, such as physical exercise (26). The effects observed are similar to those found in this study. In addition, bed rest can adversely affect musculoskeletal and cardiovascular systems and increase the risk of frailty (27). We designed the prehabilitation exercises to include three light-intensity exercises combined with isometric exercises (breathing exercises and hand grips), dynamic exercises (shoulder abduction and ankle exercises), and resistance exercises (quadriceps contraction and relaxation, curls, and lumbar exercises). These exercises are effective in improving frailty, cardiorespiratory function, emotions, and cognition (26, 28–30). The effect of different mobility exercises is different, and patients with different states of frailty may have different optimal mobility training, where patients with pre-frailty may be suitable for moderate-intensity exercises, while patients with severe frailty may only be able to perform simple prehabilitation exercises (31, 32). In addition, the type of exercise intervention may need to be tailored to the patient’s wishes and their environment (33). Currently, the optimal exercise program is unknown, and different exercise programs should be designed for different populations. One of the findings of this study was that the prehabilitation program improved the grip strength of patients after surgery, but did not improve the incidence of postoperative complications, which is similar to the conclusion of a previous study (34).

Another salient finding of this study was that prehabilitation exercise significantly improved the quality of short-term recovery after LCCS according to QoR-9 score. The QoR-9 assesses physical and mental health through five dimensions: Emotional state, physical comfort, psychological support, physical independence, and pain. Although we did not analyze the differences in each of the five dimensions in detail, the quality of recovery in the PR-ERAS group was significantly better than that in the S-ERAS group in all four postoperative assessments, confirming the advantage of prehabilitation exercises. In addition, prehabilitation exercises accelerated the recovery of gastrointestinal function after LCCS and promoted early bed removal, the result that further affirms the role of prehabilitation in improving the quality of patients’ short-term postoperative recovery. Similar to the findings of this study, previous research has found that physical exercise has beneficial effects on physical and psychosocial health (35), with particularly positive effects on depressive symptoms (36). HADS screens patients for anxiety and depression while in the hospital, and our study found that prehabilitation exercises improved patients’ anxiety and depression in the hospital. Patients in PR-ERAS group have faster time to ambulation and time to first flatus than S-ERAS group, which means patients in PR-ERAS group have faster postoperative recovery. Therefore, it can improve the patient’s postoperative experience. The factors influencing anxiety and depression may be multifaceted and related to individual personality traits, disease duration, and severity of the speed of disease recovery. Therefore, it is possible that this outcome was also influenced by the patient’s early recovery. There is evidence that physical activity improves cognitive function and reverses some effects of chronic disease (37), thereby improving surgical outcomes. But cognitive function was not assessed in this study, so we don’t know whether our prehabilitation exercise program will improve patients’ cognitive function. The focus of this study was on the effect of prehabilitation exercise on postoperative short-term outcomes in patients undergoing colorectal cancer surgery. Therefore, this study did not explore the effect of our prehabilitation protocol on patients’ long-term outcomes, including oncological features. We will follow the patients in this study for a period of 5 years and will demonstrate the effect of the prehabilitation protocol on the long-term outcome of patients undergoing colorectal cancer surgery in future studies. Previous studies have found that unsupervised outpatient prehabilitation program does not improve postoperative outcomes after elective pancreaticoduodenectomy (38). Therefore, increased compliance of prehabilitation programs is important, it can improve patient prognosis (39), and clinicians should consider not only the safety and efficacy of the protocols but also patient compliance when developing prehabilitation protocols. It is also necessary to develop appropriate prehabilitation protocols for patients according to the types of surgery. In addition, prehabilitation protocols need to be adjusted according to the physical and psychological conditions of patients, patients with good general condition can consider moderate intensity rehabilitation protocols, while patients with poor general condition should focus on light intensity prehabilitation protocols. Increased communication with patients, providing repeated education, and improved nutrition can also promote post-LCCS rehabilitation.

The present study has some limitations: Firstly, the patients were followed up with for a short period of time after surgery, it is best to wait 6-8 weeks to better observe the expected results, but most of the observations in this study lasted only until the 7th day after surgery, there were no indicators of intermediate postoperative outcomes. Secondly, due to the short follow up time, this study did not include the long-term outcomes of the patients, and we will present the long-term outcomes of the patients in future studies, including the oncological data. Thirdly, although the present study was able to demonstrate the benefit of prehabilitation exercises on the improvement of postoperative frailty, the sample size was small, and the prehabilitation protocol we explored may not be the most appropriate for patients undergoing elective LCCS. Fourthly, all patients in this study undergoing minimally invasive surgery, so the impact of the present prehabilitation protocol on postoperative frailty and the quality of recovery after open colorectal resection is debatable. Finally, power analysis was performed only for primary outcome, but not for other secondary outcomes.

## 5 Conclusion

In patients scheduled for LCCS, ERAS in combination with prehabilitation exercises, improves postoperative frailty status, enhances the quality of recovery, and accelerates the recovery of gastrointestinal function. However, it did not improve elective postoperative functional capacity or reduce the incidence of complications. Therefore, it is necessary to develop a more suitable prehabilitation protocol for patients undergoing LCCS. Additionally, improvements in compliance with prehabilitation programs and the discovery of individualized prehabilitation protocols need to be emphasized.

## Data Availability

The raw data supporting the conclusions of this article will be made available by the authors, without undue reservation.
