# Supplementary material for: Effect of prehabilitation exercises on postoperative frailty in patients undergoing laparoscopic colorectal cancer surgery
Source: Front Oncol. 2024 Sep 12;14:1411353. doi: 10.3389/fonc.2024.1411353 (PMC11424377; doi:10.3389/fonc.2024.1411353)
Supplement: Supplementary file 1 [file SupplementaryFile1.docx]

**Supplementary** **material 1**: Standard enhanced recovery after surgery (S-ERAS) protocol for elective laparoscopic colorectal resection.

The standard Enhanced Recovery After Surgery (ERAS) protocol was designed and supervised by a multidisciplinary team, including surgeons, nursing staff, and anesthesiologists.

1. Preoperative counseling, patient education, and risk assessment.

2. Nutritional assessment and enteral nutrition support.

3. Avoid preoperative mechanical bowel preparation.

4. The preoperative fasting time was6–8 hours for solid food and 2 hours for clear liquids.

5. Oral intake of 200–400 mL carbohydrate drink: up to 2–3 h before the induction of anesthesia (10% glucose solution).

6.No nasogastric tube.

7.Prophylactic intravenous antibiotic infusion 30-60 min before surgery.

8. Short-acting anesthetics were administered and EEG bispectral index monitoring was performed to maintain an appropriate depth of sedation.

9. The use of general anesthesia combined with preoperative ultrasound-guided nerve block and transversus abdominis plane (TAP) block (bilateral blocks with 20 mL of 0.5% ropivacaine) is recommended.

10.The initial CO2 flow rate was controlled at 1 L/min, and the pneumoperitoneum pressure was maintained at 8-12 mmHg according to the field exposure conditions.

11. Prevention of intraoperative hypothermia.

12. Near-zero intraoperative fluid equilibrium was maintained to avoid over- or under-volume loading.

13. Perioperative blood glucose control (80–150 mg/dL).

14. Multimodal prevention of post-operative nausea and vomiting (PONV) (5-HT3receptor antagonist + dexamethasone + droperidol).

15. Prevention of postoperative DVT (physical prophylaxis combined with low-molecular weight heparin).

16. Prevention of stress ulcers (perioperative administration of proton-pump inhibitors).

17. Multimodal management of post-operative pain (PCIA, TAP, NSAIDs, and COX-2 inhibitors).

18. Recommendations for early oral intake (drinking water 4 h after surgery, oral nutritional supplements on the first day after surgery, and a semisolid diet on the second day after surgery).

19. Recommendations for early mobilization (out-of-bed activity for 2 h on the first postoperative day and 4–6 h from the second postoperative day to discharge).

20. Abdominal drainage is not routinely placed. Unless there are risk factors for anastomotic leakage such as poor blood flow, high tension, infection, and unsatisfactory anastomosis.


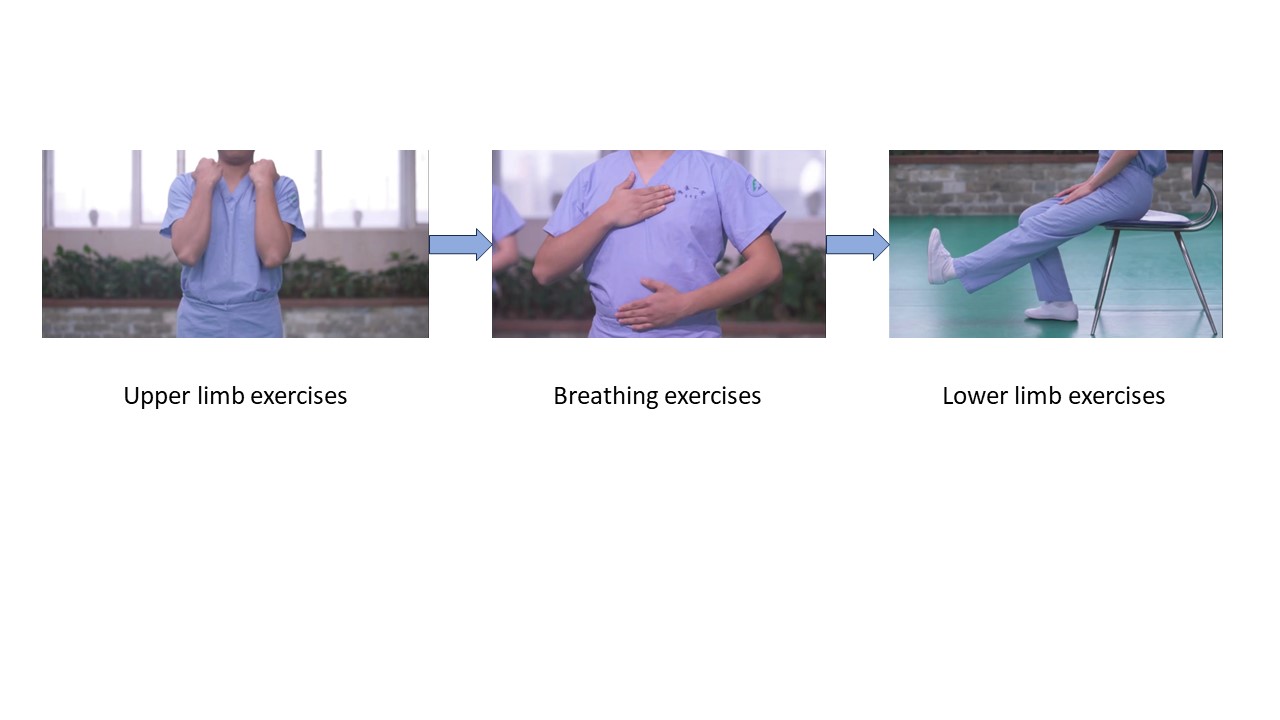


**Figure S1**. Implementation of prehabilitation exercises.

From the day the surgery was scheduled until the preoperative day, patients in the enhanced recovery after surgery based on prehabilitation group were required to perform prehabilitation exercises twice a day, in the morning and afternoon. On the day of the scheduled surgery, a rehabilitation therapist provided demonstrations and video guidance. Every patient was required to complete a diary self-report, and the rehabilitation therapist followed the patient by phone or WeChat daily before admission. The rehabilitation therapist provided bedside guidance and follow-up of patients after admission (with the assistance of a video guide).

Table S1. The difference between colectomy and proctectomy in PR-ERAS group

|  | Colectomy group (n=16) | Proctectomy group (n=34) |  |
| --- | --- | --- | --- |
| NRS2002 score | 3.5±1.2 | 3.2±1.1 | 0.34 |
| Weight loss | 7(43.8%) | 23(67.6%) | 0.434 |
| Walking time (s) |  |  |  |
| Baseline | 5±0.5 | 5.2±0.5 | 0.154 |
| 7 days after surgery | 7±1.6 | 7.2±1.8 | 0.561 |
| Exhaustion |  |  |  |
| Baseline | 1(6.3%) | 4(11.8%) | 0.753 |
| 7 days after surgery | 15(93.8%) | 31(91.2%) | 0.544 |
| Low activity |  |  |  |
| Baseline | 2(1.3%) | 6(17.6%) | 0.634 |
| 7 days after surgery | 2(1.3%) | 7(20.6%) | 0.487 |
| Grip strength (Kg) |  |  |  |
| Baseline | 26.4±8.8 | 27.6±8 | 0.609 |
| 7 days after surgery | 22±6.9 | 23.7±7.8 | 0.473 |
| Frailty Phenotype scores |  |  |  |
| Baseline | 1.1±0.9 | 0.9±0.8 | 0.277 |
| 7 days after surgery | 2.4±1.1 | 2.4±1 | 0.828 |
| HADS score |  |  |  |
| Baseline | 11.1±8.7 | 8±7.4 | 0.198 |
| 7 days after surgery | 10.1±8.7 | 8.5±7.6 | 0.511 |
| SRSS score |  |  |  |
| Baseline | 17.6±4.4 | 17±5.7 | 0.198 |
| 7 days after surgery | 18.9±4.9 | 18.1±5.7 | 0.511 |
| QoR-9 score |  |  |  |
| 1 days after surgery | 14.3±1.6 | 14.4±1.6 | 0.798 |
| 3 days after surgery | 15.4±1.5 | 15.4±1.5 | 0.961 |
| 5 days after surgery | 16.3±1.5 | 16.4±1.4 | 0.653 |
| 7 days after surgery | 17.5±6.3 | 17.7±1.6 | 0.429 |
| When scheduled surgery |  |  |  |
| 6MWD (m) | 429±68 | 455.5±65 | 0.191 |
| Borg score before 6MWD | 0(0,0) | 0(0,0.5) | 1 |
| Borg score after 6MWD | 0(0,0.5) | 0(0,0.5) | 0.153 |
| 7 days after surgery |  |  |  |
| 6MWD (m) | 248.7±52.2 | 278.3±64.4 | 0.115 |
| Borg score before 6MWD | 0(0,0) | 0(0,0) | 1 |
| Borg score after 6MWD | 0.5(0.5,1.25) | 0.5(0,1) | 0.059 |

Variables are presented as n (%), mean ± standard deviation, or median (interquartile range). NRS-2002, Nutritional Risk Screening 2002; HADS, Hospital Anxiety and Depression Scale; SRSS, Sleep Self-Rating Scale; QoR, Quality of recovery; 6MWD, 6-minute walking distance.

Table S2. The difference between PR-ERAS group with and without ostomy

|  | Ostomy group (n=17) | Non-ostomy group (n=33) |  |
| --- | --- | --- | --- |
| NRS2002 score | 3.2±1.1 | 3.3±1.1 | 0.64 |
| Weight loss | 5(29.4%) | 13(39.4%) | 0.486 |
| Walking time (s) |  |  |  |
| Baseline | 5.2±0.5 | 5.1±0.5 | 0.302 |
| 7 days after surgery | 7.6±1.9 | 6.9±1.7 | 0.227 |
| Exhaustion |  |  |  |
| Baseline | 1(5.0%) | 4(12.1%) | 0.65 |
| 7 days after surgery | 15(88.2%) | 31(94.0%) | 0.597 |
| Low activity |  |  |  |
| Baseline | 4(23.5%) | 4(12.1%) | 0.419 |
| 7 days after surgery | 3(17.6%) | 6(18.2%) | 1 |
| Grip strength (Kg) |  |  |  |
| Baseline | 26.6±8.2 | 27.6±8.3 | 0.698 |
| 7 days after surgery | 22.7±7.9 | 23.4±7.3 | 0.754 |
| Frailty Phenotype scores |  |  |  |
| Baseline | 0.8±0.7 | 1±0.9 | 0.476 |
| 7 days after surgery | 2.5±0.9 | 2.4±1.1 | 0.581 |
| HADS score |  |  |  |
| Baseline | 9.5±7.9 | 8.7±8 | 0.718 |
| 7 days after surgery | 10.6±8.7 | 8.1±7.4 | 0.287 |
| SRSS score |  |  |  |
| Baseline | 17.9±7.1 | 16.8±4.1 | 0.47 |
| 7 days after surgery | 20.1±6.4 | 17.5±4.7 | 0.112 |
| QoR-9 score |  |  |  |
| 1 days after surgery | 14.2±1.8 | 14.5±1.4 | 0.598 |
| 3 days after surgery | 15±1.8 | 15.5±1.3 | 0.212 |
| 5 days after surgery | 16±1.6 | 16.6±1.2 | 0.166 |
| 7 days after surgery | 17.6±0.7 | 17.6±0.6 | 0.698 |
| When scheduled surgery |  |  |  |
| 6MWD (m) | 450.7±68.8 | 445.1±66.1 | 0.781 |
| Borg score before 6MWD | 0(0,0) | 0(0,0) | 1 |
| Borg score after 6MWD | 0(0,0.5) | 0(0,0.5) | 0.421 |
| 7 days after surgery |  |  |  |
| 6MWD (m) | 258.1±56.5 | 274.4±64.6 | 0.384 |
| Borg score before 6MWD | 0(0,0) | 0(0,0) | 1 |
| Borg score after 6MWD | 0.5(0,1) | 0.5(0,1) | 0.975 |

Variables are presented as n (%), mean ± standard deviation, or median (interquartile range). NRS-2002, Nutritional Risk Screening 2002; HADS, Hospital Anxiety and Depression Scale; SRSS, Sleep Self-Rating Scale; QoR, Quality of recovery; 6MWD, 6-minute walking distance.
